# Delivering the cell-impermeable DNA ‘light-switching’ Ru(ii) complexes preferentially into live-cell nucleus *via* an unprecedented ion-pairing method†

**DOI:** 10.1039/c5sc03796d

**Published:** 2016-04-05

**Authors:** Ben-Zhan Zhu, Xi-Juan Chao, Chun-Hua Huang, Yan Li

**Affiliations:** a State Key Laboratory of Environmental Chemistry and Ecotoxicology, Research Center for Eco-Environmental Sciences, The Chinese Academy of Sciences P.O. Box 2871 Beijing P. R. China 100085 bzhu@rcees.ac.cn +86-10-62923563 +86-10-62849030; b Linus Pauling Institute, Oregon State University Corvallis OR 97331 USA

## Abstract

The dipyridophenazine (dppz) based ruthenium polypyridyl complexes are known as molecular ‘light-switches’ for DNA. This property is poised to serve in diagnostic and therapeutic applications, but the poor cellular uptake restricts their use in live cells. Herein, we show that the cellular uptake, and more interestingly and surprisingly, the nuclear uptake of cell-impermeable Ru(ii)–polypyridyl cationic complexes such as [Ru(bpy)_2_(dppz)]^2+^ were remarkably enhanced by three structurally unrelated biochemical agents (pentachlorophenol, carbonyl cyanide *p*-(trifluoromethoxy)phenylhydrazone and tolfenamic acid), by forming lipophilic and relatively stable ion-pair complexes, *via* a passive diffusion mechanism. Enantioselective imaging of live-cell nuclear DNA was observed between the two chiral forms of Ru(ii) complexes. This represents the first report of an unprecedented new method for delivering the DNA ‘light-switching’ Ru(ii) complexes into the nucleus of living cells *via* ion-pairing, which could serve as a promising general live-cell delivery method for other potentially bio-medically important but cell-impermeable metal complexes.

## Abstract 2

The cell-impermeable DNA ‘light-switching’ Ru(ii) complexes can be delivered into live-cell nucleus by forming lipophilic Yin–Yang ion-pairs with hydrophobic weak-acids.

## Graphical abstract

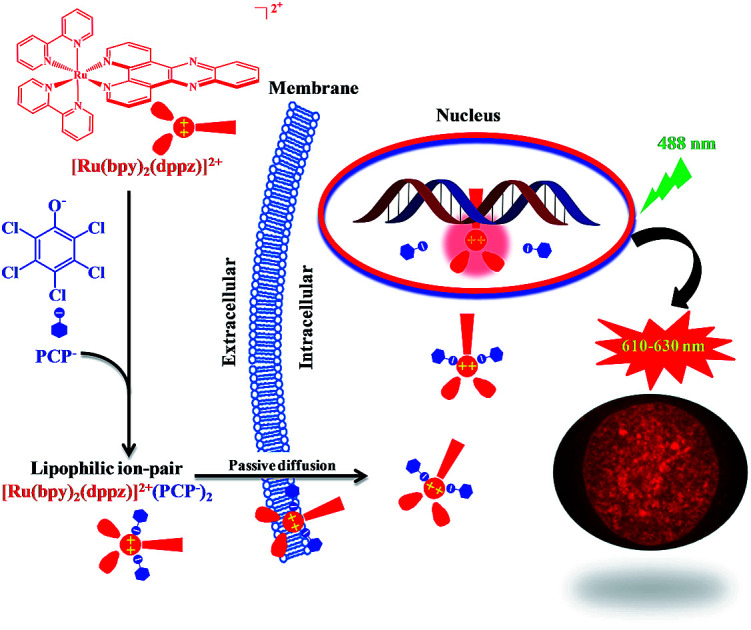

## Introduction

Since the discovery of DNA as the genetic material carrier, study towards the elucidation of DNA structure within the cell nucleus has become of great importance. Fluorescent microscopy using luminescent, cell membrane permeable organic DNA-binding molecules as probes is a well established technique towards achieving this goal.^1^ However, many of the currently available fluorescent dyes, such as DAPI (4′,6-diamidino-2-phenylindole) and Hoechst stains, suffer from narrow Stokes shift, near UV illumination, DNA photo damage and photo-bleaching.^2^

In the quest for new and better biological imaging agents, transition metal complexes provide a promising avenue for the design of diagnostic and therapeutic agents. Metal complexes able to emit from triplet metal-to-ligand charge transfer (MLCT) states offer many advantages as luminescent probes of DNA structure. Ever since it was discovered that the cationic ruthenium complex [Ru(bpy)_2_(dppz)]^2+^ (bpy = 2,2′-bipyridine, dppz = dipyrido[3,2-*a*:2′,3′-*c*]phenazine) functions as a molecular ‘light switch’ for DNA,^3^ great attention has been drawn to the DNA binding properties of polypyridyl complexes of *d*^6^ octahedral metal ions, specifically toward the development of highly sensitive and structure-specific DNA probes.^4*–*6^

As DNA imaging tools, such complexes offer several advantages over existing systems: MLCT excitations in the visible region, high Stokes shifts along with relatively low toxicity, and chemical and photo-stability. Until recently, study has been largely focused on the development of *in vitro* probes. The few studies involving direct imaging of DNA in live cells with such systems have had very limited success,^7^ with poor membrane permeability still being ascribed the major limiting factor.^8–11^

Barton's group found that the classic and structurally simplest [Ru(bpy)_2_(dppz)]^2+^ was unable to permeate into cells due to its poor lipophilicity.^8^ Strategies using either lipophilic ancillary ligands, such as 4,7-diphenyl-1,10-phenanthroline (DIP)^8^ or conjugation to protein (or steroids and peptides),^10–12^ have been employed to increase the membrane permeability of DNA-binding metal complexes. Although this has led to the successful cellular uptake of several metal-based MLCT luminescent systems,^8,10–12^ the *in cellulo* DNA binding of such systems has not been successfully demonstrated in live cells, although observed in permeabilized and fixed cells.^13,14^

During our study on the mechanism of synergistic chemical and biological effects between organic and inorganic compounds (especially metal complexes),^15–20^ we unexpectedly found that not only the cellular uptake, but more interestingly and importantly, the nuclear uptake of the cell-impermeable Ru(ii)–polypyridyl cationic complexes such as [Ru(bpy)_2_(dppz)]^2+^ were remarkably enhanced by three structurally unrelated biochemical agents, possibly *via* forming novel lipophilic and relatively stable ion-pair complexes.

## Results and discussion

### Cellular and especially nuclear uptake of [Ru(bpy)_2_(dppz)]^2+^ was greatly enhanced by pentachlorophenol (PCP)

It is well-known that the dppz complexes of Ru(ii) serve as ‘light-switches’ for non-aqueous environments, luminescing only when bound to nucleic acids, the hydrophobic regions of membranes, and other macromolecules in an aqueous solution.^3,12^ Therefore, we can use the luminescence of these Ru(ii) complexes to track their cellular and nuclear uptake by both flow cytometry and confocal laser-scanning microscopy (CLSM).

**Fig. 1:**
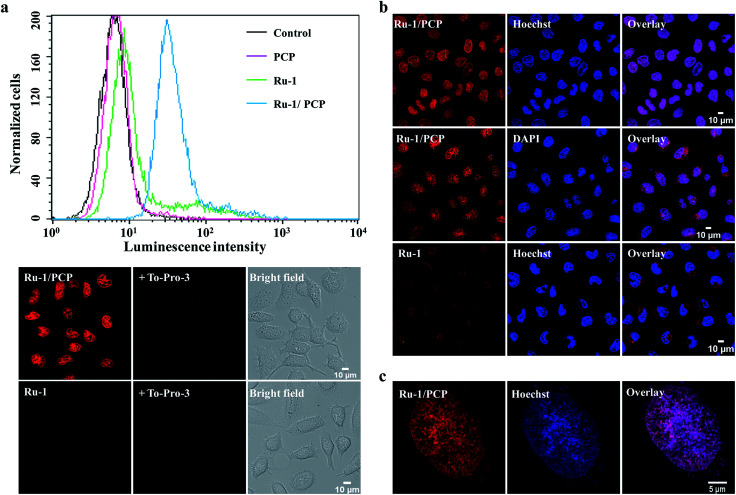
Cellular and especially nuclear uptake of [Ru(bpy)_2_(dppz)]^2+^ complex (Ru-1) was greatly enhanced by pentachlorophenol (PCP) in live cells. (a) Flow cytometry analysis (top) and confocal laser-scanning microscopy images (bottom) of QSG-7701 cells incubated with Ru-1 (100 μM) in the absence or presence of PCP (300 μM) for 3 h at 37 °C, in complete culture medium containing 10% FBS. Dead cell stain To-Pro-3 (1 μM) was used to exclude or detect dead cells. (b and c) Further confirmation that the luminescence is in the nucleus as shown by co-staining [Ru(bpy)_2_(dppz)]^2+^/PCP with the two known membrane-permeable DNA stains (DAPI and Hoechst 33342), and tracking them by both confocal microscopy (b) and the three dimensional structured illumination microscopy (SIM) with higher spatial resolution (c). Cells were incubated with Ru-1(100 μM) and PCP (300 μM) together, or with Ru-1 (500 μM) alone, for 3 h at 37 °C.

We found that not only the cellular, but more interestingly and surprisingly, the nuclear uptake of the cell-impermeable model Ru(ii)–polypyridyl cationic complex, [Ru(bpy)_2_(dppz)]^2+^, was remarkably enhanced in the presence of PCP (Fig. 1a and S1a†). PCP is a biochemical agent that has been used primarily in the protection and preservation of wood products worldwide, and for eliminating snails to prevent *schistosomiasis* in developing countries.^20,21^ As can be observed by the relative intensities of cellular luminescence at different times, as revealed by CLSM, the cellular uptake of [Ru(bpy)_2_(dppz)]^2+^ also gradually increases with time when present together with PCP (Fig. S1b†). Quantitation by line plots indicates nuclear uptake of intense luminescence in the nucleus compared to other regions (Fig. S1c†).

To further confirm that the luminescence is in the nucleus, we co-stained [Ru(bpy)_2_(dppz)]^2+^/PCP with the two known membrane-permeable DNA stains (DAPI and Hoechst 33342) and a RNA stain (SYTO 9), and tracked them by both CLSM and the three dimensional structured illumination microscopy (SIM) with higher spatial resolution. The almost complete overlay images clearly show the nuclear DNA stain of [Ru(bpy)_2_(dppz)]^2+^ in the presence of PCP (Fig. 1b and c). It should be emphasized that the cells were alive after all these treatments (Fig. S2†) (see Experimental section for details on how to determine the health of the cells).

**Fig. 2:**
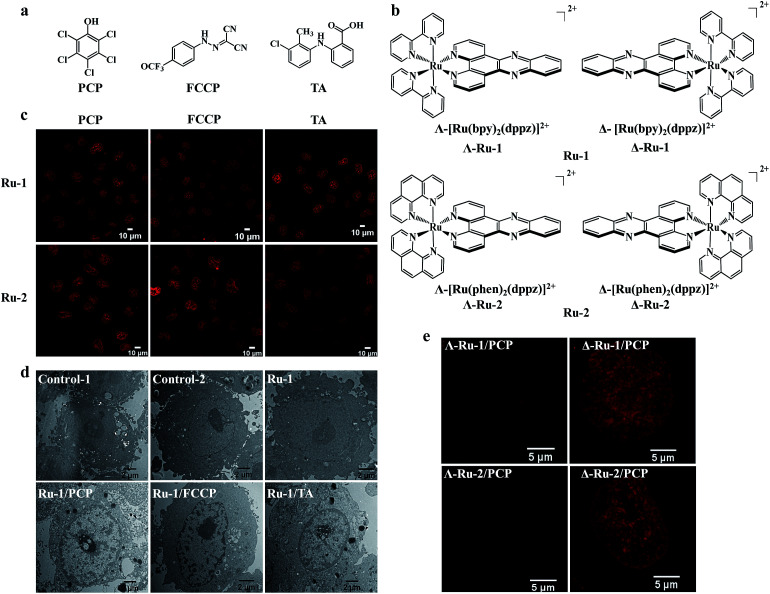
Nuclear uptake of Ru(ii) complexes was enhanced by three structurally unrelated biochemical agents and the enantioselective imaging of live-cell nuclear DNA by the two chiral forms of Ru(ii)–dppz complexes. Chemical structures of the three biochemical agents (a) and the enantiomers of Ru(ii)–dppz complexes (b). (c) Confocal images of cells treated with Ru-1 (100 μM) or Ru-2 (100 μM) in the presence of PCP (300 μM) for 3 h in complete medium, Ru-1 (200 μM) or Ru-2 (500 μM) in the presence of FCCP (50 μM) for 1 h in serum-free medium, and Ru-1 (300 μM, 3 h) or Ru-2 (100 μM, 4 h) in the presence of TA (300 μM) in complete medium. (d) Cellular localization of Ru-1, characterized by transmission electron microscopy (TEM) in the absence or presence of PCP, FCCP, and TA for 3 h. Negative control cells stained with nothing (control-1) or solely with osmium tetroxide (control-2); cells incubated with Ru-1 (1 mM) alone, Ru-1 (300 μM)/PCP (300 μM), Ru-1 (500 μM)/FCCP (100 μM), Ru-1 (500 μM)/TA (300 μM) and additionally stained with osmium tetroxide. (e) Dramatic differences were observed between the two chiral forms (Δ and Λ) of Ru-1 and Ru-2 in live-cell nucleus DNA imaging, with Δ-enantiomers showing much brighter emission intensity inside the nucleus compared to Λ-enantiomers. Structured illumination microscopy images of cells incubated with Δ-Ru (100 μM) or Λ-Ru (100 μM) in the presence of PCP (300 μM) in cells for 3 h.

In addition to these luminescence microscopy studies, which rely on the DNA binding and subsequent activation of the ‘light-switch’ effect to observe the *in cellulo* location of [Ru(bpy)_2_(dppz)]^2+^, we were also able to show the nuclear distribution of [Ru(bpy)_2_(dppz)]^2+^ in live cells using transmission electron microscopy (TEM), with better spatial resolution^7^ (while organic dyes cannot be observed with this method). We found that [Ru(bpy)_2_(dppz)]^2+^ was clearly incorporated into the nucleus of cells in the presence of PCP, which is consistent with CLSM and SIM studies (Fig. 2d).

In clear contrast, cells treated with [Ru(bpy)_2_(dppz)]^2+^ complex (0.1 mM) alone for a short time (3 h) showed only a minor change in the luminescence profile. At higher concentration (0.5 mM) and even after longer incubation time (24 h), the [Ru(bpy)_2_(dppz)]^2+^ complex could be taken up by cells, but only in the cytoplasm, not in the nucleus (Fig. S3a†).

Similar nuclear uptake results were observed when the QSG-7701 cell was substituted with other cell-lines, including HeLa, HepG-2, HL-7702, MCF-7 and PC-12 cells, as well as bacteria such as *Staphylococcus aureus* (Fig. S3b†).

Taken together, the complementary application of flow cytometry, live-cell CLSM, SIM coupled with co-staining and TEM studies demonstrated unequivocally that [Ru(bpy)_2_(dppz)]^2+^ were not only taken up by live eukaryotic and prokaryotic cells in the presence of PCP, but more importantly, they were readily delivered into the nucleus of living cells.

### The enhanced cellular and nuclear uptake is a general phenomenon for all cationic Ru(ii) polypyridyl complexes and two other structurally unrelated biochemical agents

It is interesting to know whether the enhanced cellular and nuclear uptake of [Ru(bpy)_2_(dppz)]^2+^ is only limited to PCP, or if it is a general phenomenon for other structurally different groups of biochemical agents. Because PCP is a hydrophobic weak acid (log *K*_ow_, 5.1; p*K*_a_, 4.7), with a bulky aromatic pentachlorophenyl group, we wondered whether other biochemical agents with similar physiochemical characteristics could function similarly. To our great surprise, we found that the cellular and nuclear uptake of [Ru(bpy)_2_(dppz)]^2+^ were markedly enhanced, not only by PCP, but also by carbonyl cyanide *p*-(trifluoromethoxy)phenylhydrazone (FCCP), a classic and potent uncoupler of oxidative phosphorylation in mitochondria,^22^ and more importantly, by tolfenamic acid (TA) (Fig. 2c and d), a member of the fenamic acid class of the non-steroidal anti-inflammatory drugs (NSAIDs) currently used in the treatment of migraine attacks.^23^ It should be noted that although the three compounds (PCP, FCCP and TA) belong to three structurally unrelated biochemical agents (Fig. 2a), they possess the following common biochemical characteristics: they are all weak acids with a bulky aromatic hydrophobic moiety and strong electron-withdrawing substituents.

In addition to [Ru(bpy)_2_(dppz)]^2+^, other cationic Ru(ii) polypyridyl complexes, including the typical [Ru(phen)_2_(dppz)]^2+^ (phen = 1,10-phenanthroline), [Ru(phen)_3_]^2+^ and [Ru(bpy)_3_]^2+^, have also been extensively studied.^4,24^ We found that their cellular and nuclear uptakes were all enhanced by PCP, FCCP and TA (Fig. 2c, and S4a†).

The abovementioned results suggest that the enhanced cellular and nuclear uptake, with three structurally unrelated hydrophobic weak acids, is a general phenomenon for all cationic Ru(ii) polypyridyl complexes.

### Enantioselective imaging of live-cell nuclear DNA was observed between the two chiral forms (Δ- and Λ-enantiomer) of Ru(ii)–dppz complexes

It should be noted that the Ru(ii) complexes were used as racemic mixtures in the abovementioned studies. The use of chiral metal complexes to probe the structure of DNA has been an active area of research for over half a century. The chiral Ru(ii)–dppz complex enantiomers, with their characteristic propeller shapes, have been successfully used as diastereomeric probes to discriminate between right- and left-handed duplex DNA in isolated DNA.^25,26^ Therefore, the chirality of the Ru(ii)–dppz complexes should be another very important feature for an *in cellulo* DNA structural probe. Interestingly, we found that there is a dramatic difference between the two chiral forms (Δ- and Λ-enantiomers) of [Ru(bpy)_2_(dppz)]^2+^ and [Ru(phen)_2_(dppz)]^2+^ in live-cell imaging of DNA nucleus (Fig. 2b and e), with Δ-enantiomers showing much brighter emission intensity inside the nucleus compared to Λ-enantiomers. The original intuition that a Δ-handed octahedral Ru(ii)–dppz complex must be a better fit for the right-handed DNA duplex is visualized here for the first time in living cells, more than 60 years after the original double helix model was proposed.^27^ Interestingly, these results in live-cell nucleus correspond well with that in pure isolated DNA^25^ and in methanol-fixated cells.^14^ The higher quantum yield of the DNA-bound Δ-enantiomer might be due to its longer excited-state lifetimes than the DNA-bound Λ-enantiomer, which arise from an overall more favorable binding geometry.^25,26^

### Molecular mechanism of the enhanced cellular and nuclear uptake of Ru(ii) complexes by PCP: the formation of neutral, lipophilic and relatively stable ion-pair complexes

It is well known that there are four main pathways for cellular uptake, including endocytosis, active transport, facilitated diffusion and passive diffusion. One clear dividing line among the different mechanisms is whether uptake requires energy (endocytosis and active transport proteins), or is energy-independent (passive diffusion through the membrane and diffusion facilitated by carriers). Pathways, such as endocytosis and active transport, can be blocked by incubating cells at low temperature (4 °C) or by ATP-depletion with metabolic inhibitors, such as 2-deoxyglucose (competitively inhibits glycolysis) and oligomycin (blocks oxidative phosphorylation),^5^ otherwise, if uptake of the test compound is unchanged, the mechanism is an energy-independent process. In our study, we found that treatment with 2-deoxyglucose and oligomycin did not reduce the cellular uptake of [Ru(bpy)_2_(dppz)]^2+^ in the presence of the three different biochemical agents (here PCP was chosen as a representative) (Fig. S5b†), suggesting an energy-independent mode of entry. Because the most common energy-dependent pathway by which eukaryotic cells take up materials is endocytosis, we further investigated the cellular uptake of [Ru(bpy)_2_(dppz)]^2+^/PCP by co-incubating cells with several well-known inhibitors of this process. As can be observed by the relative intensities after treatment with these inhibitors, neither the general endocytosis inhibitors (ammonium chloride or chloroquine diphosphate), nor the specific endocytosis inhibitor (chlorpromazine hydrochloride) had any effect on the uptake. Moreover, colchicine, which is known to disrupt the microtubule polymerization, also showed no inhibition of uptake (Fig. S5c†). However, in contrast to the energy-dependent pathway, the cellular uptake of [Ru(bpy)_2_(dppz)]^2+^/PCP shows temperature-dependent transport (Fig. S5a†), which is probably because higher temperature increases membrane fluidity and solubility of [Ru(bpy)_2_(dppz)]^2+^.

It is well known that organic cation transporters (OCT) can facilitate the diffusion of endogenous organic cations, as well as a variety of drugs and toxins.^5^ The possible role of an OCT was then explored. We found that [Ru(bpy)_2_(dppz)]^2+^ uptake is not significantly affected in cells co-incubated with the OCT inhibitor cimetidine (Fig. S5d†), indicating that OCT is not responsible for the cellular uptake of [Ru(bpy)_2_(dppz)]^2+^.

With all the inhibitors used above, the results suggest that passive diffusion should be the major mechanism of cellular uptake of [Ru(bpy)_2_(dppz)]^2+^ in the presence of PCP. Passive diffusion is less cell-type specific, allows greater freedom for modification of the complex than transport *via* membrane proteins, and does not lead to entrapment in endosomes, as often occurs with endocytosis. As a result, this mechanism of passive diffusion may portend the broad applicability of metal complexes in different cell types for different intracellular functions (see above, Fig. S3b†).

**Fig. 3:**
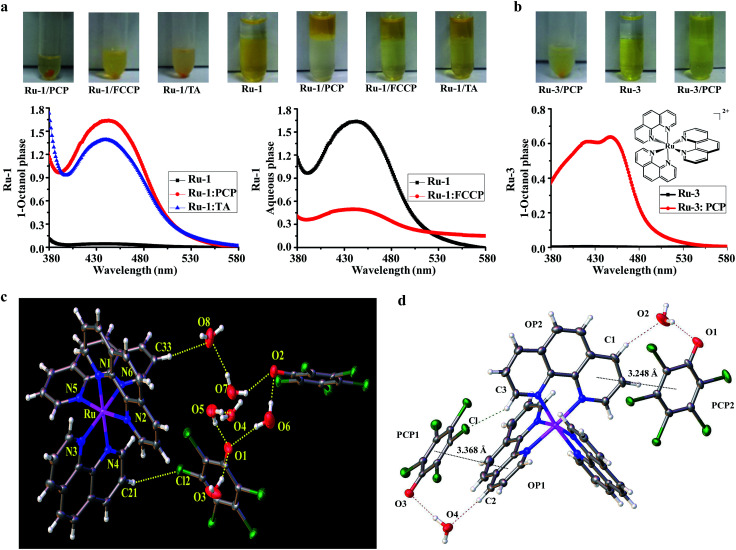
Evidence for the formation of neutral and lipophilic ion-pair complexes between Ru(ii) complexes and the three biochemical agents. (a) Precipitation of Ru-1 and PCP, FCCP, TA, in aqueous buffer solution; partition studies of Ru-1 (100 μM) between 1-octanol and aqueous phases (tris–HCl buffer, 10 mM, pH 7.4) in the absence or presence of PCP (1 mM), FCCP (1 mM), and TA (1 mM). (Note: because both FCCP and Ru-1 have similar strong absorption spectra, to avoid their interference with each other, the spectra were obtained in aqueous phase in the case of FCCP). (b) Precipitation between Ru-3 and PCP in aqueous buffer solution, and partition studies of Ru-3 (100 μM) in the absence or presence of PCP (1 mM). (c) Crystal structure of the ion pair complex [Ru(phen)_3_]^2+^(PCP^−^)_2_ by ORTEP drawing with 30% thermal ellipsoids, the H-bonds are indicated by dotted lines. (d) Crystal packing diagram showing stacking interactions between [Ru(phen)_3_]^2+^ cation and PCP^−^ anion within the [Ru(phen)_3_]^2+^(PCP^−^)_2_ complex. The black dotted lines indicate the π⋯π interactions between the PCP ring and the parallel phen ring; the red dashed line indicates the cation–anion C–H⋯(H_2_O)⋯O hydrogen bonds; the green dashed line indicates the cation–anion C–H⋯Cl hydrogen bond. The other solvent molecules were omitted for clarity.

Because lipophilicity has been considered an important factor in the cellular uptake of metal coordination complexes,^8,9^ we speculate that a lipophilic adduct might be formed between the hydrophilic and cell-impermeable [Ru(bpy)_2_(dppz)]^2+^ and PCP, thus readily penetrating through cell cytoplasmic membranes. Indeed, we found that an oily red droplet was formed and precipitated when PCP was added to the yellow [Ru(bpy)_2_(dppz)]^2+^ in an aqueous buffer solution (pH, 7.4) (Fig. 3a), suggesting a new adduct may be formed between them. To test whether this new adduct is lipophilic or not, 1-octanol/aqueous partitioning study was conducted, because the 1-octanol/water partitioning system is the common reference system for the determination of lipophilicity, widely employed for structure–activity studies. We found as expected that [Ru(bpy)_2_(dppz)]^2+^ alone cannot be partitioned into the organic phase, because it has a low partition coefficient (log *P* = −2.50)^8^ (Fig. 3a). However, when PCP was present, [Ru(bpy)_2_(dppz)]^2+^ was readily partitioned into the 1-octanol phase (Fig. 3a). These results indicate that a lipophilic adduct was indeed formed between [Ru(bpy)_2_(dppz)]^2+^ and PCP. Analogous effects were observed with other Ru(ii) polypyridyl complexes and PCP, FCCP and TA (Fig. 3b, and S4b–e†). Furthermore, there arose the question of the binding nature of this lipophilic adduct.

Ion pairing has been considered to be one of the most fundamental atomic interactions in both chemistry and biology. Ion pairs were visualized as associations of two oppositely charged ions, retaining their basic properties when bonded together by coulombic forces, and to a lesser extent, by other interactions.^28,29^ Chemists have been fascinated by ion pairs for some time, particularly when one or more of the ions is a transition metal coordination compound.^29^ Such ion pairs have practical importance in catalysis and chromatography, as well as in some types of batteries and solar cells.^28–30^

The electric neutrality of the ion-pairs makes them non-conducting and sometimes lipophilic; therefore, they can be extracted into organic solvents or penetrate through cytoplasmic membranes.^31,32^ Ion-pairing reactions have often been utilized by coupling with solvent extraction and HPLC analysis, which have been used mainly for the separation and the concentration of ionic analytes. Although it has been assumed that ion pairing may also play an important role in biological system such as in ionic drug delivery, there have been very few convincing examples to date, especially in the case of hydrophilic metal cationic complexes.^31,32^

Since [Ru(bpy)_2_(dppz)]^2+^ is a coordinatively saturated and substitutionally inert, positively-charged cationic complex, and PCP is a weak hydrophobic acid that could be readily dissociated to produce its negatively-charged pentachlorophenolate anion under physiological pH, we speculate that a neutral and lipophilic ion pair complex might be formed between the two oppositely charged bulky aromatic ions. If this were true, the chemical composition of the ion pair should be [Ru(bpy)_2_(dppz)]^2+^[PCP^−^]_2_. However, we found that the typical analytical methods used for solutions were not suitable for structure determination of the unknown, mainly because of its poor solubility in both water and organic solvents. We also tried to co-crystallize it, but unfortunately, our efforts were not successful (possibly due to the presence of the phenazine nitrogens of the dppz ligand).

Because PCP was also found to enhance the cellular and nuclear uptake of other Ru(ii) complexes such as [Ru(phen)_3_]^2+^ (a close analog of [Ru(bpy)_2_(dppz)]^2+^), we then extended our study to the interactions between [Ru(phen)_3_]^2+^ and [PCP^−^]. Fortunately, a single crystal could be cultivated after incubation of [Ru(phen)_3_]^2+^ and [PCP^−^], and its solid-state structure was determined using single-crystal X-ray diffraction. The solution of the diffracted data clearly showed that an ion pair was indeed formed between [Ru(phen)_3_]^2+^ and [PCP^−^], with a 1 : 2 stoichiometry (Fig. 3c; for detailed crystal data, see Tables S1 and S2†). The ion pair [Ru(phen)_3_]^2+^[PCP^−^]_2_ should belong to the outer sphere ion-pair that represents contact ion-pairs, in which the coordinatively saturated first coordination sphere of the cation is no longer accessible to the anion, and as a consequence, the anion is relegated to the second coordination sphere, interacting with the cation through electrostatic and other weak forces only (aromatic pi stacking, H-bonding, δ–δ, and CH–δ) (Fig. 3d). We assume that a similar ion pair complex should be formed between [Ru(bpy)_2_(dppz)]^2+^ and [PCP^−^], and indeed, we found that the binding affinity between [Ru(bpy)_2_(dppz)]^2+^ and the three biochemical agents (PCP, FCCP and TA) are relatively strong, as measured by the fluorescence displacement method using calf thymus DNA (Table S3 and Fig. S6†) (see Experimental sections for details on how to measure binding affinity).

**Scheme 1:**
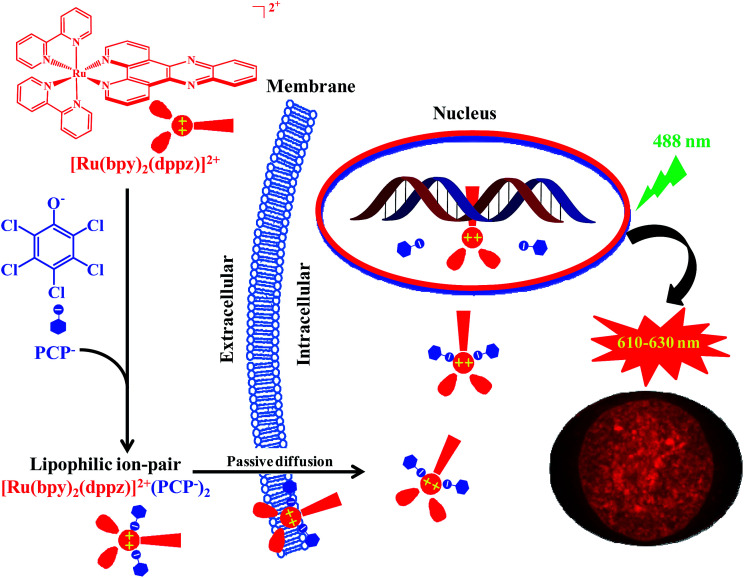
Proposed molecular mechanism for delivering the cell-impermeable DNA ‘light-switching’ Ru(ii) complexes by PCP into the nucleus in live cells, by forming lipophilic and relatively stable ion-pair complexes *via* a passive diffusion mechanism.

The most interesting and surprising finding of this study is that PCP can enhance not only cellular uptake, but more importantly, it can also deliver the Ru(ii) complex directly into the nucleus while maintaining the DNA recognition characteristics of the parent coordination complex (Fig. S6†). The reason for this and the possible underlying mechanism were investigated. We propose that it can be readily explained by the formation of not only neutral and lipophilic ion-pairs, but also relatively strong and stable ion-pair complexes between [Ru(bpy)_2_(dppz)]^2+^ and PCP (Scheme 1). The ion pair [Ru(bpy)_2_(dppz)]^2+^[PCP^−^]_2_ should be strong and stable enough (Table S3†) to compete with various binding components present either in cell culture medium (such as 10% FSB) or in cytoplasm, and lipophilic enough (Fig. 3) to penetrate through cytoplasmic and nuclear membranes. At the same time, it should be weak and labile enough to be readily dissociated, once it reaches nuclear DNA (that has stronger binding affinity for [Ru(bpy)_2_(dppz)]^2+^ (*K* = ∼10^6^),^3^ and the dissociated [Ru(bpy)_2_(dppz)]^2+^ would then bind tightly with nuclear DNA (possibly *via* intercalation).^33^ The “freed” PCP may diffuse back to the medium, and then transport more [Ru(bpy)_2_(dppz)]^2+^ into the cell, possibly serving as a shuttle carrier.

This is in clear contrast to other studies aimed at effecting membrane permeability by increasing hydrophobicity through coupling to bigger molecules to form conjugates.^10–12^ While changes in hydrophobicity *via* conjugation can modulate cellular uptake, they can also affect either cellular localization of the parent complex, which often leads to a decrease in nuclear targeting, or the characteristics of luminescence of the parent complex.^10–12^ The study outlined herein shows that such an approach is not always required and represents a significant step in the development of these DNA binding systems toward *in vivo* applications.

### What is unique for this study and its potential biological implications?

Intracellular transport of different biologically active molecules, including various positively or negatively charged metal complexes, is one of the key problems in drug delivery in general. Another problem is that even after being safely delivered into the cell cytoplasm, drugs still have to find their way to specific organelles (nuclei, lysosomes, and mitochondria), wherein they are expected to utilize their therapeutic and diagnostic potential. This is especially important in the case of DNA-targeting metal complexes. However, the lipophilic nature of the biological membranes restricts the direct intracellular delivery of such positively charged hydrophilic metal complexes. The methods, such as microinjection or electroporation, used for the delivery of membrane-impermeable molecules in cell experiments, are invasive in nature and could damage the cellular membranes.^34,35^ If the positively-charged metal complexes were neutralized by the negatively-charged hydrophobic weak acids to form lipophilic and relatively stable ion-pair complexes, then the abovementioned problems should be readily solved.

In this study, we found that the enhanced cellular uptake, and especially nuclear uptake of the cell-impermeable cationic Ru(ii) complexes can be achieved *via* forming lipophilic and relatively stable ion-pair complexes with three structurally unrelated hydrophobic weak acids. We suggest that the formation of ion pair complexes with similar lipophilic character and stability could be of relevance as a general mechanism for the delivery of other potentially bio-medically important, but cell-impermeable metal complexes into the expected cellular targets.

Compared with two other conventional DNA stains (Hoechst 33342 and DAPI) in living cells, [Ru(bpy)_2_(dppz)]^2+^/PCP has the following remarkable advantages as a fluorescence probe (Table 1): [Ru(bpy)_2_(dppz)]^2+^ is highly water soluble, photostable (Fig. S7†) and displays a large Stokes shift value with a long lifetime far-red emission, a factor that makes it extremely compatible with other imaging agents such as the heavily used green fluorescent protein (GFP). It is also well tolerated by both eukaryotic and prokaryotic cells, and more importantly, it can be used for both luminescence and TEM studies, which is one of the most distinguished characteristics of this system. Lastly, we found that there is a dramatic difference between the two chiral forms of [Ru(bpy)_2_(dppz)]^2+^ and [Ru(phen)_2_(dppz)]^2+^, therefore the chirality of the Ru(ii) complex is another very important feature as an *in cellulo* DNA structural probe.

**Table 1:** Ru(ii)–dppz complexes/PCP can be used as live-cell DNA probes with many unsurpassed characteristics, compared with the current commonly used organic DNA probes^a^

	Ru(ii)–dppz^a^/PCP	DAPI	Hoechst 33342
Excitation/emission maximum (nm)	445/620	358/461^b^	350/461^b^
Stokes shift	>150	103	111
TEM	+	−	−
Photo-bleaching	Low	High (1–2 min)	High
UV damage	−	+	+
Enantioselectivity	+	−	−
Cell permeable	+	−/+	+
Living cell/fixed cell	+/+	+/+	+/+
Binding mode	Intercalator^c^	MGB/AT preference^b^	MGB/AT preference^b^
Working concentration	80 μg ml^−1^	0.5–10 μg ml^−1^	0.25–20 μg ml^−1^

a Ru(ii)–dppz: [Ru(bpy)_2_(dppz)]^2+^ and [Ru(phen)_2_(dppz)]^2+^.

b Molecular probes.

c Ref. 33.

Ru(ii)–dppz complexes have been used in a wide variety of applications, including DNA detection, DNA topoisomerase and RNA polymerase inhibition, DNA footprinting, amyloid-β-fibrillisation and α-synuclein aggregation detection, cell imaging, photodynamic therapy, anti-proliferative and anticancer effects.^4–14,25,26^ However, almost all these applications have not yet been realized in living cells. Because there are huge differences between non-cellular and live-cell systems, luminescent Ru(ii) complexes capable of passive cell delivery may find a huge number of potential *in cellulo* or *in vivo* applications in those areas, which may lead to more interesting new findings.

## Conclusions

In conclusion, we report for the first time, that the cell-impermeable Ru(ii) polypyridyl ‘light switch’ complexes were readily taken up by cells and preferentially localized in the nucleus in the presence of the three structurally unrelated biochemical agents. This was carried out by the formation of lipophilic and relatively stable ion-pairs by a passive diffusion mechanism, which can be used as a live cell DNA probe with many unsurpassed characteristics, compared to the currently commonly used organic DNA probes. Ion pairing could serve as a promising, general, live-cell delivering method for other potentially bio-medically important, but cell-impermeable metal complexes.

## Supplementary Material

SC-007-C5SC03796D-s001

SC-007-C5SC03796D-s002
