# Supplementary material for: Delivering the cell-impermeable DNA ‘light-switching’ Ru(ii) complexes preferentially into live-cell nucleus via an unprecedented ion-pairing method
Source: Chem Sci. 2016 Apr 5;7(7):4016–23. doi: 10.1039/c5sc03796d (PMC6013783; doi:10.1039/c5sc03796d)
Supplement: SC-007-C5SC03796D-s001 [file SC-007-C5SC03796D-s001.pdf]

Electronic Supplementary Material (ESI) for Chemical Science.
This journal is © The Royal Society of Chemistry 2016

Supplementary Information (SI) for

Delivering the Cell-impermeable DNA 'Light-switch' Ru(II) Complexes Preferentially into Live-cell Nucleus via an Unprecedented Ion-Pairing Method**

Ben-Zhan Zhu*, Xi-Juan Chao, Chun-Hua Huang and Yan Li

Contents

Experimental Sections……………………………………………………………………………………..3

References ……………………..…………….……………………………..……………………………………4

Figure S1 - Figure S7…………………………………………………...………………………………..…..5 - 10

Table S1 - Table S3………………………..……………………………………………………………...11 –12

Experimental Sections

Chemicals

The $[Ru(bpy)_2(dppz)]^{2+}Cl_2$ and $[Ru(phen)_2(dppz)]^{2+}Cl_2$ complexes were synthesized according to references,[1-3] $[Ru(bpy)_3]^{2+}Cl_2$ and $[Ru(phen)_3]^{2+}Cl_2$ were purchased from Sigma-Aldrich. Pentachlorophenol (PCP), carbonyl cyanide *p*-(trifluoromethoxy)phenyl-hydrazone (FCCP) and tolfenamic acid (TA) were purchased from Sigma. To-Pro-3 (TP3) was purchased from Invitrogen. Oligomycin, 2-deoxy-D-glucose, and all other inhibitors were purchased from Sigma-Aldrich.

Cell Culture

QSG-7701, HepG2, HL-7702 cells were cultured in RPMI 1640 medium with 10% fetal bovine serum, MCF-7, HeLa cells were cultured in DMEM medium with 10% fetal bovine serum (FBS), PC-12 cells were cultured in DMEM medium with 6% heat inactivated horse serum and 6% FBS, all with 1% penicillin-streptomycin, at 37 °C under a 5% CO_2 atmosphere. *Staphylococcus aureus* were grown in nutrient broth medium, pH 7.4, 37 °C.

Flow Cytometry

QSG-7701 cells were detached from monolayer culture with trypsin, re-suspended in medium with serum, and diluted to 1×10^6 cells/ml. The $[Ru(bpy)_2(dppz)]^{2+}$ complex and PCP were added to the cell suspensions at concentrations of 100 μM and 300 μM, respectively, and incubated for 3 h. The cells were isolated by centrifugation and rinsed with cold PBS (phosphate buffered saline) for 3 times, dead cells were stained with To-Pro-3 (1 μM), which enters cells with compromised membranes. Temperature dependence studies used cells that had been cooled at 4 °C and incubated with 100 μM Ru(II) complexes at 4 °C for 3 hour. For inhibitors, cells were previously treated with NH_4Cl, chlorpromazine and colchicine at the stated concentrations for 30 minutes, and then incubated with Ru(II) complexes/PCP for 3 hours before imaging. Flow cytometry was performed on a BD FACS Caliber using ~20,000 cells per sample. Luminescence data were obtained by excitation at 488 nm with emission at 600-630 nm for Ru(II) complexes and excitation at 633 nm and observed at 650-670 nm for To-Pro-3.

Methods for Determining Cell Health

To determine the health of the cells after treatments with various chemical agents, a standard live/dead staining procedure using two commercially available dyes—SYTO 9 and To-Pro-3—was performed. SYTO 9 is a membrane-permeable nucleic acid dye, which stains the RNA of living cells. Trypan blue stain assay was used to determine whether the plasma membrane permeability was affected by PCP alone or PCP/Ru(II) complex. To further confirm whether the membrane integrity was compromised by PCP or not, two membrane-impermeable DNA dyes: To-Pro-3 and PI were also used to co-incubate with PCP.

Confocal Laser Scanning Microscopy (CLSM) and Structured Illumination Microscopy (SIM)

After incubated with Ru(II) complexes and PCP, FCCP, TA, cells were rinsed with PBS for 3 times, and were luminescently imaged on a CLSM using 40×oli-immersion lens for slide imaging, or on a super-resolution SIM using 100×oli-immersion lens. The imaging were excited at 488 nm and emission monitored at 600-630 nm. All cells were washed with PBS before imaging. Microscopy was performed on a Leica TCS SP5 CLSM or a super-resolution microscopy Delta Vision OMX SIM, respectively. Live cells were distinguished by their low To-Pro-3 emission with excitation at 633 nm and observing at 650-670 nm.

Transmission Electron Microscopy (TEM)

For TEM, cells were incubated with $[Ru(bpy)_2(dppz)]^{2+}$ (300 μM) /PCP (300 μM, 3 h), $[Ru(bpy)_2(dppz)]^{2+}$ (500 μM)/TA (300 μM, 3 h), $[Ru(bpy)_2(dppz)]^{2+}$ (500 μM)/FCCP(100 μM, 3h) then fixed with 2.5% glutaraldehyde and dehydrated using ethanol. TEM samples were sectioned in Araldite resin by microtome (Lecia EM UC6) and examined on a H-7650B instrument operating at 80 kV equipped.

Partition Study

1-Octanol/aqueous (Tris-HCl buffer, 10 mM, pH 7.4) phase partition for Ru(II) complexes in the presence or absence of PCP, FCCP and tolfenamic acid, respectively, were conducted using the "shake-flask" method, with the concentration in each phase determined by UV-vis absorbance (Beckman DU-800). Since FCCP has very similar absorption with the Ru(II) complexes, FCCP would interfere with the UV-vis spectra of Ru (II) complexes in the 1-octanol phase, so the partition experiments with FCCP were measured in aqueous phase instead.

Separation of Enantiomers

The enantiomers of $[Ru(bpy)_2(dppz)]^{2+}$ and $[Ru(phen)_2(dppz)]^{2+}$ were separated using a CYCLOBOND I 2000 DMP high-performance liquid chromatography (HPLC) column (Sigma) on a Agilent 1260 HPLC, with an isocratic solvent composition of 40/8/52 (v/v/v) 20 mM KPF_6 (aq.): CH_3CN : ETOH for $[Ru(bpy)_2(dppz)]^{2+}$ and 40/25/35 (v/v/v) 20 mM KPF_6 (aq.): CH_3CN : ETOH for $[Ru(phen)_2(dppz)]^{2+}$. The

Δ-enantiomer eluted first, followed by the Λ-isomer. Assignment of the two fractions was confirmed by circular dichroism.[4] The chloride salt of the compound was precipitated from a solution of 0.50 g of the KPF_6 in 10 ml of acetone by the addition of an acetone solution of tetra-n-butylammonium chloride. The solids were filtered off, washed with acetone and diethyl ether, dried.[5]

Cultivation of Crystals

50 μl $[Ru(II)(phen)_3]Cl_2$ (100 mM, in acetonitrile) and 100 μl PCP (100 mM, in 200 mM NaOH) were mixed in the PB buffer (pH 7.4). After 10 min of vigorous stirring, the resultant brown precipitate was filtered, then washed with 4:1 water/acetonitrile and dried in vacuum. The clean precipitate was then re-dissolved in 3:1 1, 4-dioxane/H_2O. After 3 days, single crystals suitable for X-ray analysis were grown at room temperature.

Single Crystal X-ray Diffraction

The synthetic complex was determined by single crystal X-ray diffraction. Data collection was performed on an Agilent Gemini A Ultra diffractometer, using graphite-monochromated Cu *Kα* radiation (λ = 1.5418 Å) for the complex. The determination of crystal class and unit cell parameters was carried out by CrysAlis (Agilent 2011) program package, raw frame data was processed using CrysAlis (Agilent 2011), and the structures were solved by use of SHELX97 program and refined by full-matrix least-squares on F values.

Measurement of the Binding Affinity between $[Ru(bpy)_2(dppz)]^{2+}$ and the Three Biochemical Agents

The binding affinity was measured by fluorescence displacement method using ctDNA. As we know, in aqueous solution, $[Ru(bpy)_2(dppz)]^{2+}$ complex luminesce brightly only when bound to DNA. So $[Ru(bpy)_2(dppz)]^{2+}$ can be used as fluorescent probe here due to the light-switching effect upon interaction with DNA. When chemicals compete to bind $[Ru(bpy)_2(dppz)]^{2+}$, the luminescence of $[Rubpy)_2(dppz)]^{2+}$-DNA will decrease. In the binding solution (10 mM Tris-HCl, pH 7.4), ctDNA and $[Ru(bpy)_2(dppz)]^{2+}$ were kept at 200 μM and 50 μM, respectively. Each chemical was added into the pre-incubated DNA/$[Ru(bpy)_2(dppz)]^{2+}$ solution with varying concentrations. The fluorescence signal was measured after incubation at RT for 10 min. The 50% inhibitory concentration of each biochemical agent (IC_{50}) could be obtained from the established competitive titration curve. The dissociation constants and association constants between each biochemical agent and $[Ru(bpy)_2(dppz)]^{2+}$ were calculated by the following formula[6]:

$$K_d = [IC_{50}]/(1+[probe]/K_{probe}) \qquad (1)$$

$$K_b = 1/K_d \qquad (2)$$

Where [probe] is the concentration of ctDNA, the intrinsic ctDNA binding constant *K* of $[Ru(bpy)_2(dppz)]^{2+}$ is ~10^6 M^{-1},[7] here we use 10^6 M^{-1} for calculation. K_{probe} is the dissociation constant for the intercalation of Ru in ctDNA ($K_{probe} = 1.0\times10^{-6}/ M^{-1}$).

References

1 E. Amouyal, A. Homsi, J. C. Chambron and J. P. Sauvage, *J. Chem. Soc. Dalton.*, 1990, 1841-1845.
2 B. P. Sullivan, D. J. Salmon and T. J. Meyer, *Inorg. Chem.*, 1978, **17**, 3334-3341.
3 M. Yamada, Y. Tanaka, Y. Yoshimoto, S. Kuroda and I. Shimao, *Bull. Chem. Soc. Jpn.*, 1992, **65**, 1006-1011.
4 J. G. Liu, B. H. Ye, Q. L. Zhang, X. H. Zou, Q. X. Zhen, X. Tian and L. N. Ji, *J. Biol. Inorg. Chem.*, 2000, **5**, 119-128.
5 C. Hiort, P. Lincoln and B. Norden, *J. Am. Chem. Soc.*, 1993, **115**, 3448-3454.
6 Y. Cheng and W. H. Prusoff, *Biochem. Pharmacol.*, 1973, **22**, 3099-3108.
7 A. E. Friedman, J. C. Chambron, J. P. Sauvage, N. J. Turro and J. K. Barton, *J. Am. Chem. Soc.*, 1990, **112**, 4960-4962.

SI Figures and Tables

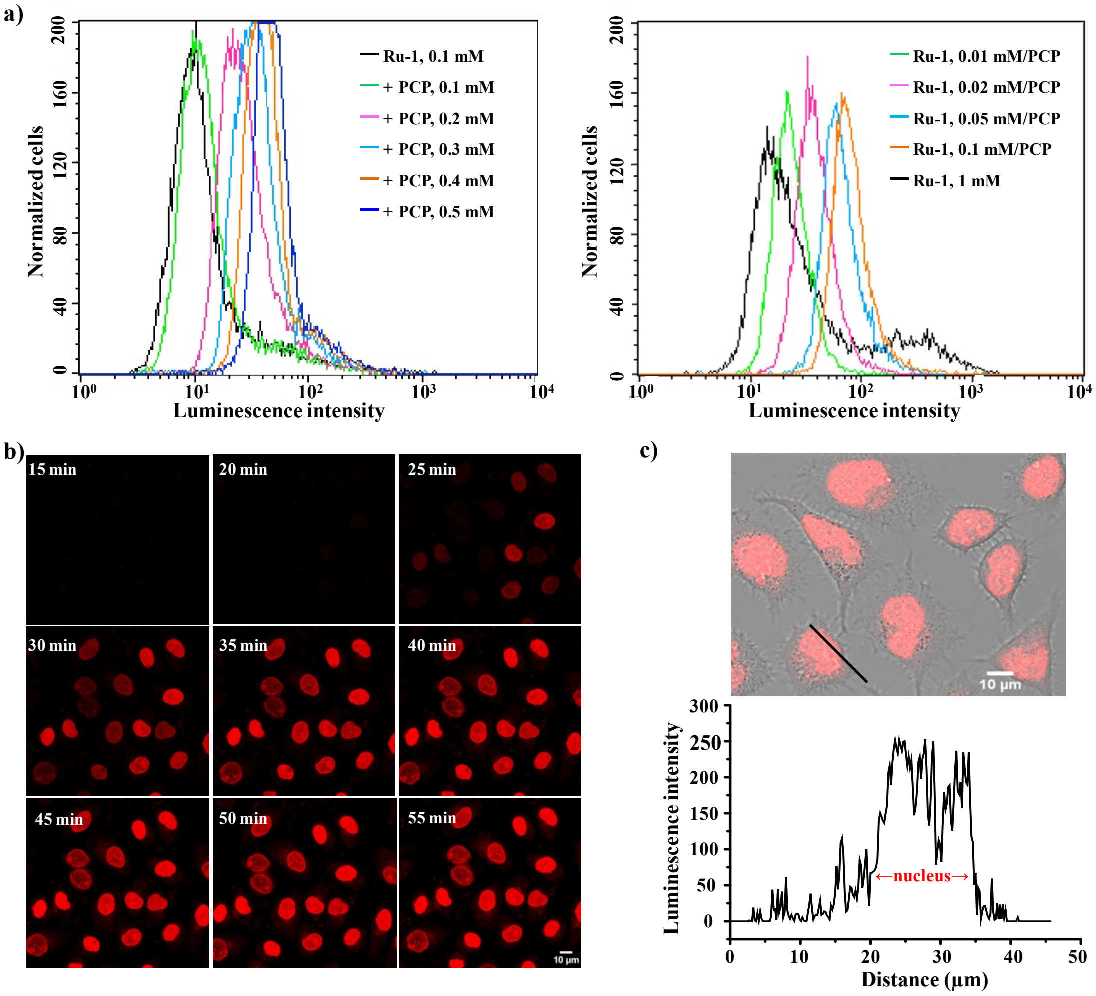

Figure S1: Dose-dependent, time-dependent and distribution of cellular and nuclear uptake of [Ru(bpy)$_2$(dppz)]$^{2+}$ (Ru-1) in the presence of pentachlorophenol (PCP) in live cells. **a**) (left) Cells incubated with Ru-1 (100 μM) and varying concentration of PCP (100, 200, 300, 400, 500 μM) for 3 h. (right) Cells incubated with PCP (300 μM) and varying concentration of Ru-1 (10, 20, 50, 100 μM) for 3 h. Ru-1 (1,000 μM) alone was used as a control. **b**) Confocal microscopy images of QSG-7701 cells incubated with Ru-1 (100 μM) and PCP (300 μM) captured per 5 min. **c**) Confocal microscopy of QSG-7701 cells incubated with Ru-1 (100 μM)/PCP (300 μM) for 3 h (top). Intensity profile of ruthenium luminescence across a cell (bottom).

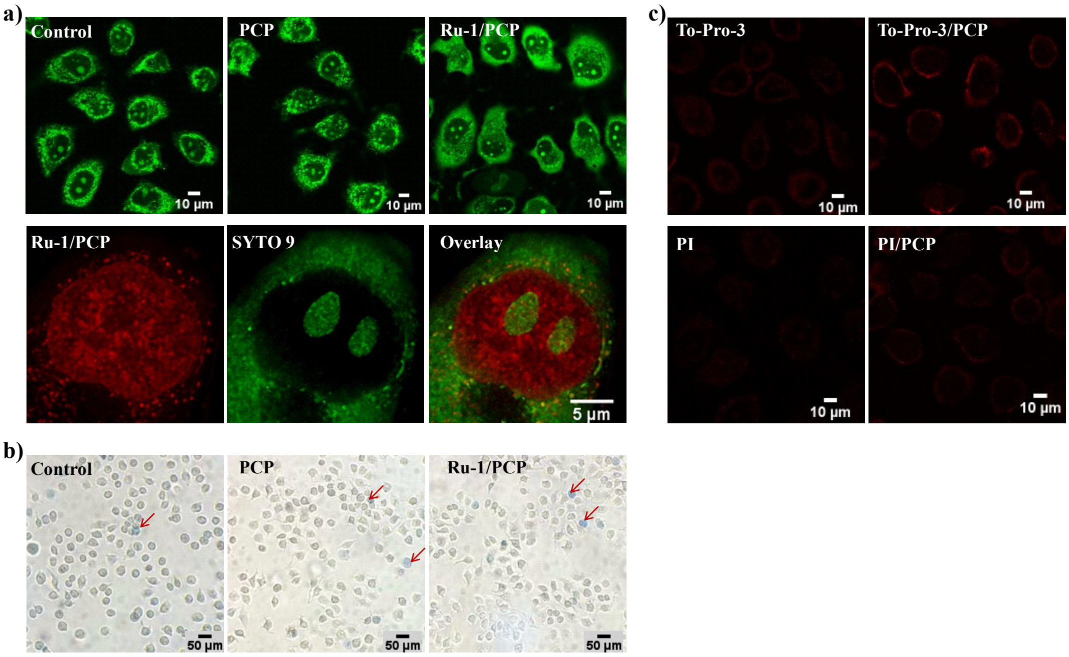

Figure S2*: *The health of cell as determined by different methods. **a**) Cells treated with PCP (300 μM) or Ru-1 (100 μM)/PCP (300 μM) for 3 h by confocal microscopy (top), and cell treated with Ru-1 (100 μM)/PCP (300 μM) for 3 h by SIM (bottom), then stained by SYTO 9 (1 μM). The observed green luminescence by SYTO 9 shows that cells are alive. **b**) Cells treated with PCP (300 μM) or Ru-1 (100 μM)/PCP (300 μM) for 3 h, and trypan blue stain assay was used to determine the membrane permeability, the results indicated membrane permeability wasn't affected by PCP. **c**) To further confirm the membrane permeability wasn't affected by PCP, two membrane impermeable DNA dyes: To-Pro-3 (1 μM) and PI (10μg/ml) were also used to co-incubate with PCP (300 μM) for 3 h, the results showed either To-Pro-3 or PI didn't get through the membrane to the nucleus, which further indicated PCP didn't affect membrane permeability and proved cellular uptake of Ru-1 wasn't a result from increased membrane permeability by PCP.

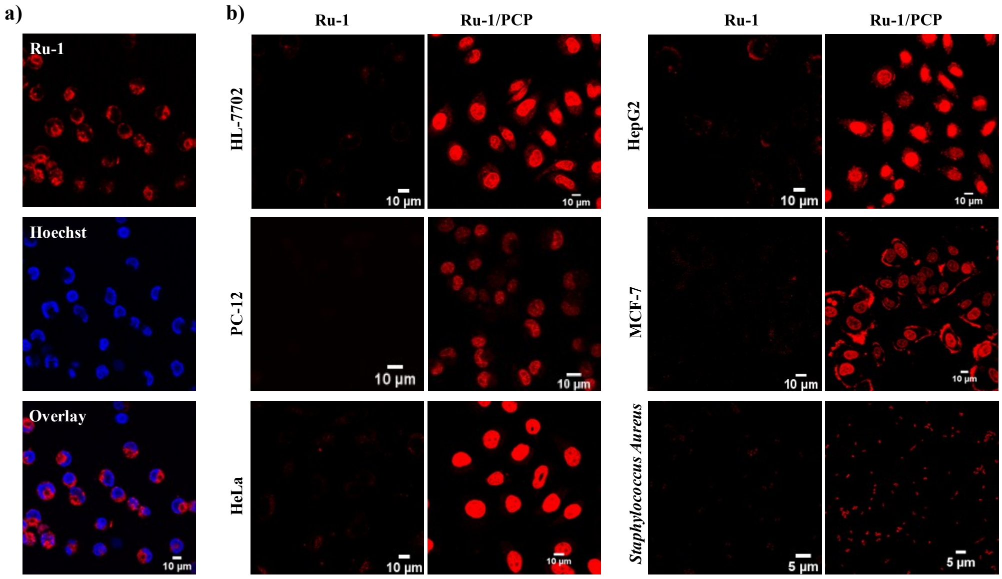

Figure S3: The localization of Ru-1 alone in QSG-7701 cells and nuclear uptake of Ru-1 in the absence or presence of PCP in several more different cell lines. **a**) Cells incubated with Ru-1 (500 μM) alone for 24 h, co-staining with Hoechst33342 shows no nuclear uptake. **b**) Cells incubated with Ru-1 (100 μM) or Ru-1 (100 μM)/PCP (300 μM) for 3 h in complete medium in different cell lines.

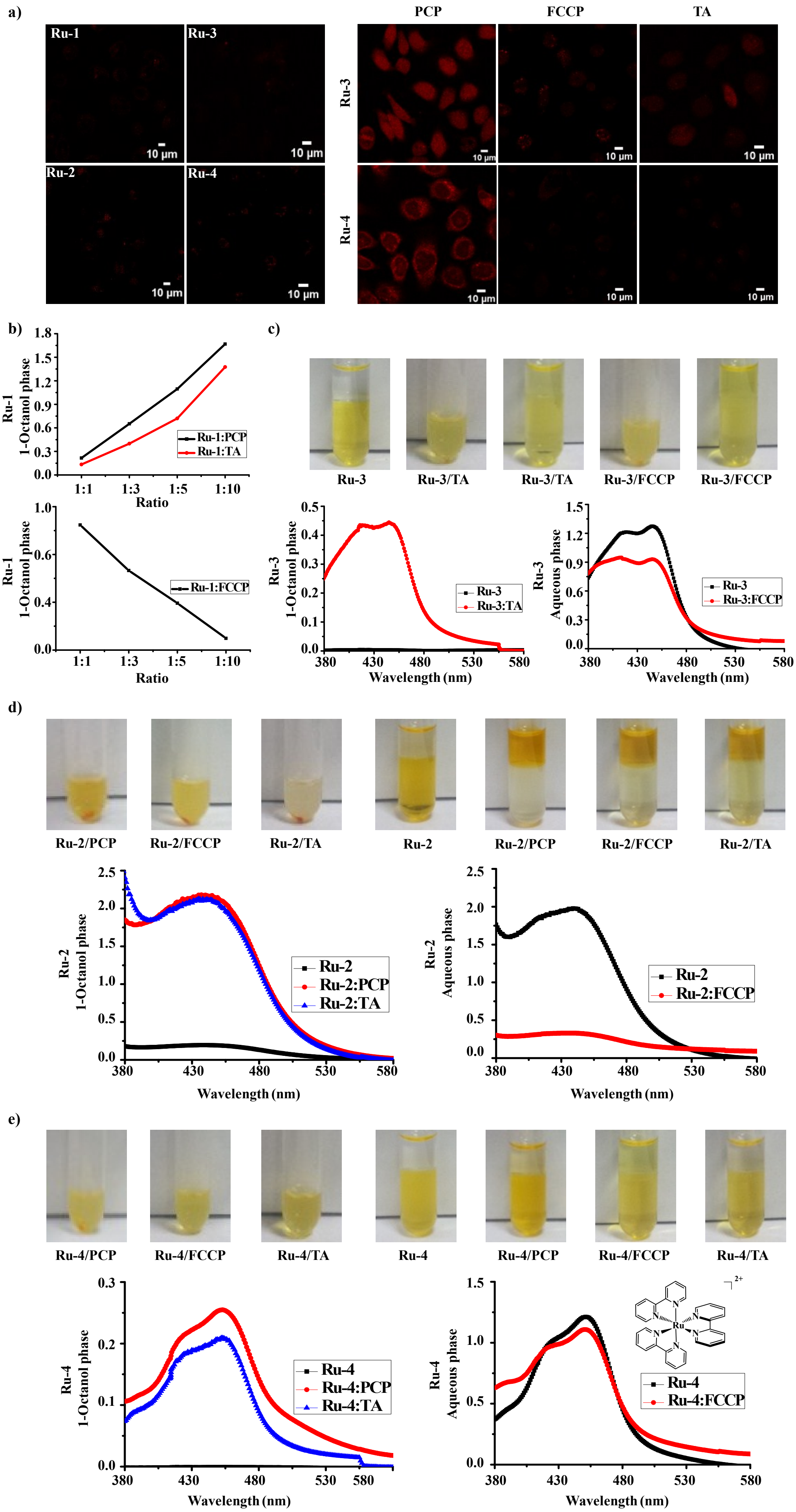
a)
Ru-1
Ru-3
Ru-2
Ru-4
10 μm
PCP
FCCP
TA
b)
Ru-1 1-Octanol phase
Ru-1:PCP
Ru-1:TA
Ru-1:FCCP
Ratio
1:1
1:3
1:5
1:10
c)
Ru-3
Ru-3/TA
Ru-3/FCCP
Ru-3 1-Octanol phase
Ru-3 Aqueous phase
Ru-3:TA
Ru-3:FCCP
Wavelength (nm)
d)
Ru-2/PCP
Ru-2/FCCP
Ru-2/TA
Ru-2
Ru-2 1-Octanol phase
Ru-2 Aqueous phase
Ru-2:PCP
Ru-2:TA
Ru-2:FCCP
e)
Ru-4/PCP
Ru-4/FCCP
Ru-4/TA
Ru-4
Ru-4 1-Octanol phase
Ru-4 Aqueous phase
Ru-4:PCP
Ru-4:TA
Ru-4:FCCP

Figure S4: Cellular uptake of [Ru(phen)$_3$]$^{2+}$ (Ru-3) and [Ru(bpy)$_3$]$^{2+}$ (Ru-4) complexes in the presence of the three biochemical agents and partition between 1-octanol and aqueous phase for Ru(II) complexes in the absence or presence of the three biochemical agents: Evidence for the formation of neutral and lipophilic ion-pair complexes. **a**) (left) Cells treated with Ru complexes (100 μM) alone for 3 h. (right) Confocal images of cells treated with Ru-3 (100 μM), Ru-4 (100 μM) in the presence of PCP (300 μM) in complete medium for 3 h; with Ru-3 (500 μM), Ru-4 (500 μM) in the presence of FCCP (50 μM) in serum-free medium for 1 h; and with Ru-3 (100 μM), Ru-4 (100 μM) in the presence of TA (300 μM) in serum-free medium for 4 h. **b**) Partition studies of Ru-1 in the presence of PCP, FCCP, TA, respectively, with different ratio. **c**) Precipitation between Ru-3 and TA, FCCP, in aqueous buffer solution. Partition studies of Ru-3 (100 μM) in the absence or presence of 1 mM TA, FCCP. **d**), **e**) Precipitation between Ru-2 (**d**) or Ru-4 (**e**) and PCP, FCCP, TA, respectively, in aqueous buffer solution. Partition studies of Ru-2 or Ru-4 (100 μM) between 1-octanol and aqueous phases (Tris-HCl buffer, 10 mM, pH 7.4) in the absence or presence of 1 mM PCP, FCCP, and TA, respectively. **Note**: Ru with FCCP was detected in aqueous phase for the high distribution of FCCP in 1-octanol and the maximum wavelength for FCCP is too closed to that for Ru(II) complexes.

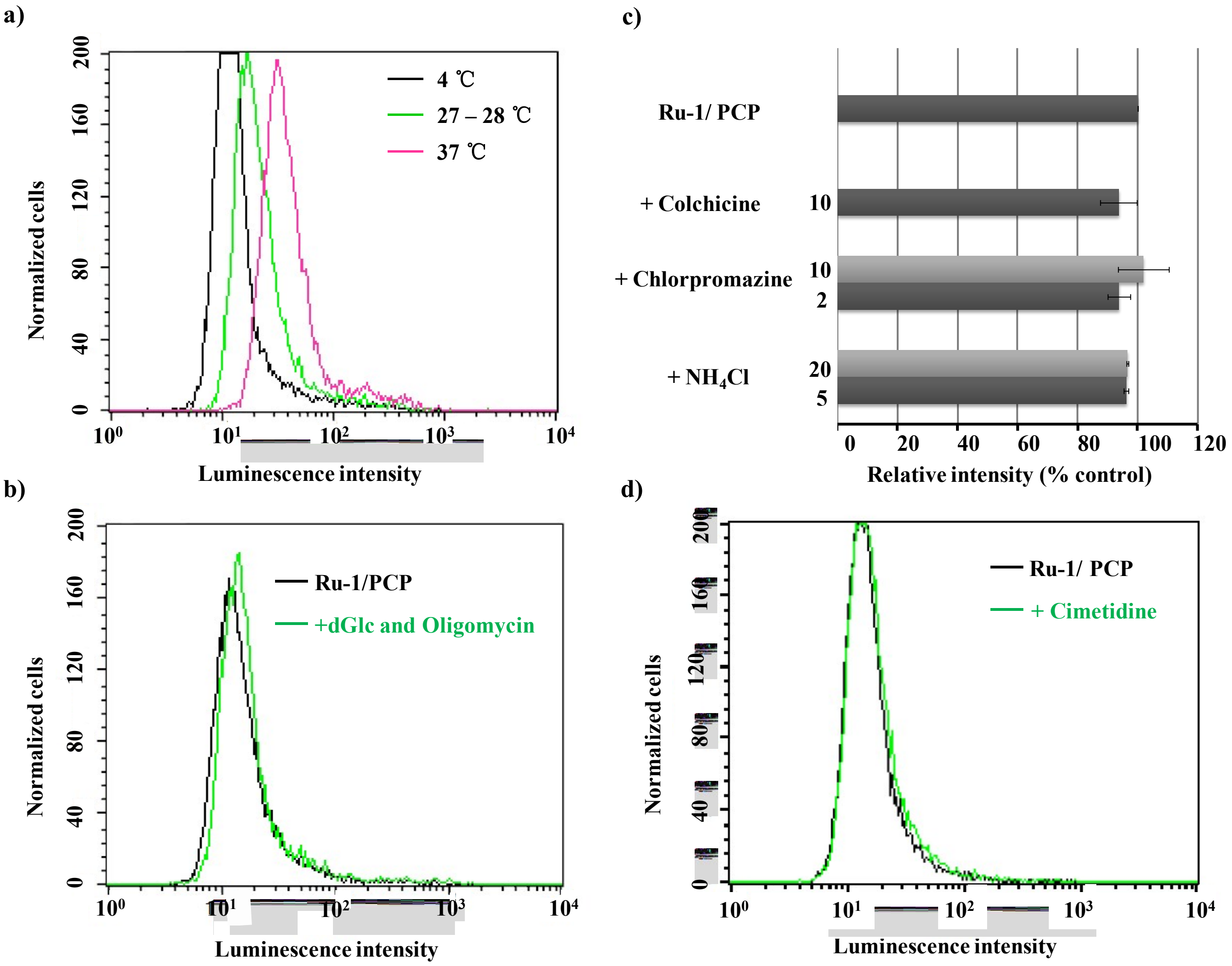

Figure S5: Mechanism of cellular uptake of Ru-1 complex in the presence of PCP. **a**) Temperature-dependence studies for cellular uptake of Ru-1 (100 μM) in the presence of PCP (300 μM) for 3 h. **b**) Luminescence intensity of Ru-1 (100 μM)/PCP (300 μM) in cells after metabolic inhibition with 2-deoxy-D-glucose (dGlc) (10 mM) and oligomycin (5 μM), 37 °C. **c**) Relative luminescence intensity of Ru-1 (100 μM)/PCP (300 μM) after endocytosis inhibition treatment with colchicine (10 μM), chlorpromazine (10 μg/ml), and NH_4Cl (20 mM), respectively. Error bars represent standard error of the mean with n = 3. **d**) Luminescence intensity of Ru-1 (100 μM)/PCP (300 μM) in cells after treatment with cimetidine (500 μM), 37 °C, an organic cation transporter inhibitor.

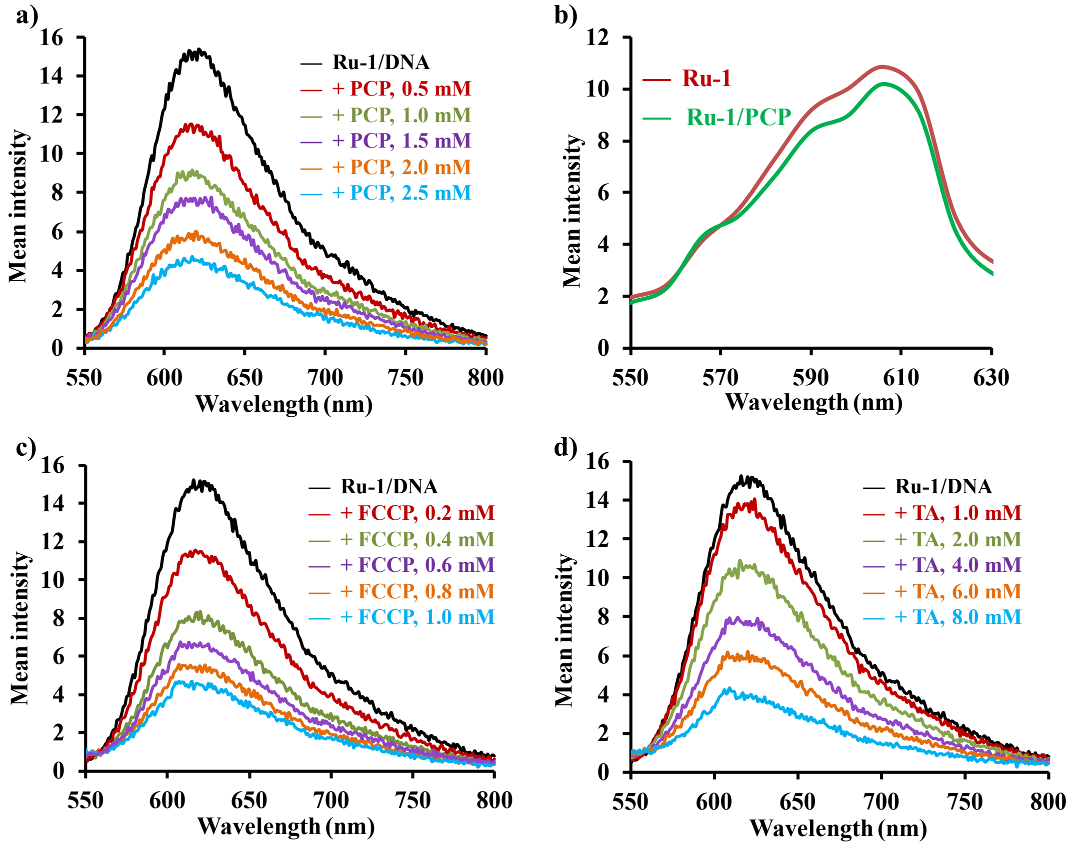

Figure S6: The photophysical properties of Ru-1 in the absence and presence of the three biochemical agents. **a)** Emission spectra of Ru-1 (50 μM)/DNA (200 μM) in the absence and presence of PCP in aqueous chemical system with excitation by 488nm. b) Intracellular emission spectra for either Ru-1 alone or Ru-1/PCP using the Lambda scanning mode of confocal. Spectrum were recorded after cells treated with Ru-1 (500 μM) and Ru-1 (100 μM)/PCP (300 μM) for 3 h. Emission spectra of Ru-1 (50 μM)/DNA (200 μM) in the absence and presence of FCCP **(c)** or TA **(d)** in aqueous chemical system with excitation by 488nm.

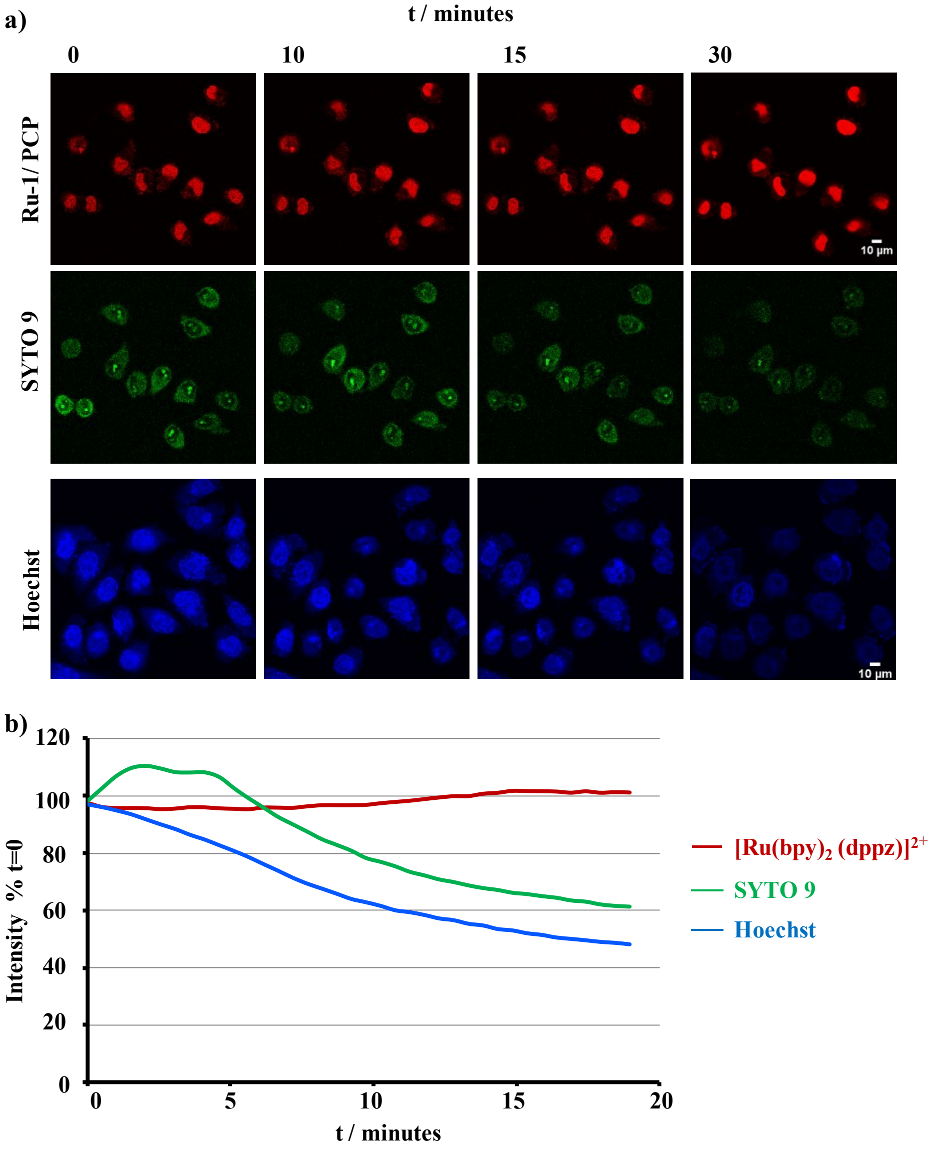

FigureS 7: Photo-stability of Ru-1/PCP compared with SYTO 9 and Hoechst33342 in cells. **a**) Time series showing luminescence of Ru-1 (100 μM) and PCP (300 μM) in cells under constant laser exposure with no observable photo-bleaching over 30 minutes exposure time (excited with 488 nm) (top, red). In contrast to SYTO 9 (1 μM), which demonstrates faster fading than Ru-1/PCP (middle, green). The same experiment was conducted with Hoechst33342 (1 μg/mL) (bottom, blue) (excited with 405 nm). **b**) Relative *in cellulo* luminescence intensity profile time series of Ru-1/PCP (red) demonstrating stronger photo-stability over SYTO 9 (green) and Hoechst33342 (blue).

Table S1: Selected distances [Å] and angles [°] of the $[Ru(phen)_3]^{2+}(PCP^-)_2$ complex

Coordination bonds			
Ru-N(1)	2.071(4)	Ru-N(4)	2.081(4)
Ru-N(2)	2.059(2)	Ru-N(5)	2.062(4)
Ru-N(3)	2.076(5)	Ru-N(6)	2.053(4)
N(1)-Ru-N(2)	79.01(16)	N(3)-Ru-N(4)	79.43(17)
N(5)-Ru-N(6)	80.36(16)		

Hydrogen bonds	d(D···A)	∠D-H···A
O3-H···O1	2.949(5)	148.9
O5-H···O1	2.740(4)	168.9
O5-H···O4	2.81	154.4
O6-H···O1	2.809(5)	170.3
O6-H···O2	2.895(5)	162.9
O7-H···O2	2.726(5)	148.2
O7-H···O8	2.813	138.2
C21-H···Cl2	3.612(6)	130.9
C33-H···O8	3.520(6)	170.9

Table S2: Crystal data of the $[Ru(phen)_3]^{2+}(PCP^-)_2$ complex

Compound	$[Ru(phen)_3^{2+}](PCP^-)_2 \cdot CH_3CN \cdot 6H_2O$
Empirical formula	$C_{50}H_{39}Cl_{10}N_7O_8Ru$
CCDC number	1046781
Formula weight	1321.45
Temperature (K)	180.15
Crystal system	orthorhombic
Space group	$Pna2_1$
a (Å)	16.7707(5)
b (Å)	12.8886(3)
c (Å)	24.1907(6)
α (°)	90
β (°)	90
γ (°)	90
Volume ($Å^3$)	5228.8(2)
Z	4
Dcalc (Mg/m^3)	1.679
Absorption coefficient (mm^{-1})	0.873
F(000)	2664
Crystal size (mm^3)	0.15 × 0.1 × 0.1
[illegible] range (°)	5.912-52.042
h, k, l ranges	-20 20, -15 15, -29 29
Reflections collected	51902
Data/restraints/parameters	10230/1/674
Goodness-of-fit on F^2	1.088
Final R indexes [I>2σ (I)]	R_1 = 0.0314, wR_2 = 0.0755
Final R indexes [all data]	R_1 = 0.0353, wR_2 = 0.0783
Largest diff. peak/hole (e. $Å^{-3}$)	0.82/-0.46

Table S3: The binding affinity between Ru-1and the three biochemical agents

Chemicals	IC_{50} /mM	K_d/M	K_b/M^{-1}
PCP	1.344	6.69×10^{-6}	1.49×10^{5}
FCCP	0.492	2.45×10^{-6}	4.08×10^{5}
TA	4.078	2.03×10^{-5}	4.93×10^{4}
